# Chopper Stabilized Low Resistance Comparator

**DOI:** 10.3390/s90402491

**Published:** 2009-04-09

**Authors:** Radojle M. Radetic, Dragan R. Milivojevic

**Affiliations:** 1 Serbian Transmission System / Nade Dimic 40, 19210 Bor, Serbia; E-Mail: rradetic@ptt.rs; 2 Mining and Metallurgy Institute, Department of Informatics / Zeleni Bulevar 35, 19210 Bor, Serbia

**Keywords:** Low resistance, measuring, chopper comparator, high accuracy

## Abstract

The paper describes an improvement of the chopper method for elimination of parasitic voltages in a low resistance comparison and measurement procedure. The basic circuit diagram along with a short description of the working principle are presented and the appropriate low resistance comparator prototype was designed and realized. Preliminary examinations confirm the possibility of measuring extremely low voltages. Very high accuracy in resistance comparison and measurement is achieved (0.08 ppm for 1,000 attempts). Some special critical features in the design are discussed and solutions for overcoming the problems are described.

## 1. Introduction

Standard methods for measurement and low resistance comparison are based on Thompson (Kelvin) bridges, DC current comparators, potentiometers, etc [1]. The new generation high quality multimeters can also be used for this kind of measurement [2,3]. In this paper an original low resistance comparator design is presented.

**Figure 1.**
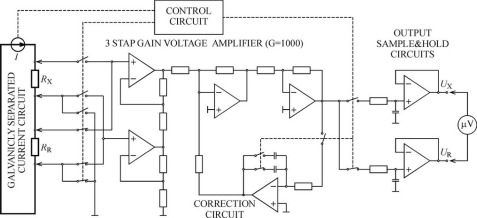
Circuit diagram of chopper stabilized comparator.

The circuit diagram of the proposed chopper stabilized comparator is given in Figure 1. The current flow through two serial connected resistors (measured *R*_X_ and reference *R*_R_) provides the adequate voltages in them, *U_X_* and *U_R_*, respectively. As known, the voltage ratio on the resistor terminals is equal to their resistances ratio. In that case, the value of measured resistance *R*_X_ could be expressed as:

(1) $$R_{X}=R_{R}\;\frac{U_{X}}{U_{R}}$$

The maximum relative measurement error is:

(2) $$\left|{\delta R}_{X}\right|=\left|\frac{{\Delta R}_{R}}{R_{\mathrm{RT}}}\right|+\left|\frac{{\Delta U}_{X}}{U_{\mathrm{XT}}}\right|+\left|\frac{{\Delta U}_{R}}{U_{\mathrm{RT}}}\right|=\left|{\delta R}_{R}\right|+\left|{\delta U}_{X}\right|+\left|{\delta U}_{R}\right|$$

where *δR*_R_ is reference resistance error (could be less than 10^−6^) and *δU*_X_, *δU*_R_ are measurement errors for the appropriate voltages.

In low resistance measurement (four-terminal resistors [4]), the voltage on the potential terminals should theoretically be *RI*, but in a real measurement environment, except for the *RI* value, parasitic voltages can occur, such as thermo-electric, noise etc. All these unwanted influences can cause measurement errors and need to be eliminated.

Thermo-electric or Peltier voltage is generated at the thermocouple junctions of different metals. Even when all the junctions are at the same temperature, the thermoelectric voltage can reach a value of about 0.1μV/°C. The most significant disturbances are a consequence of offset voltages of the operational amplifiers and can be higher than 50 μV. These are direct current (DC) parasitic voltages. Alternating current (AC) unwanted voltages can also occur. The AC parasitic voltages are a consequence of AC power supply inductive or capacitive influence, noise, etc. AC parasitic voltages cause dispersion of measured results around the mean value. The mains influence (inductive and capacitive) is periodic and can be efficiently decreased (shielding, filtering, etc). Noise is a random occurrence with a zero mean and its disturbance may be reduced to acceptable levels by filtration. DC parasitic voltages cause systematic measurement errors and need to be removed from the measurement voltage as much as possible. One solution for minimization of the influence of these parasitic voltages is presented in this paper.

## 2. Results and Discussion

**Figure 2.**
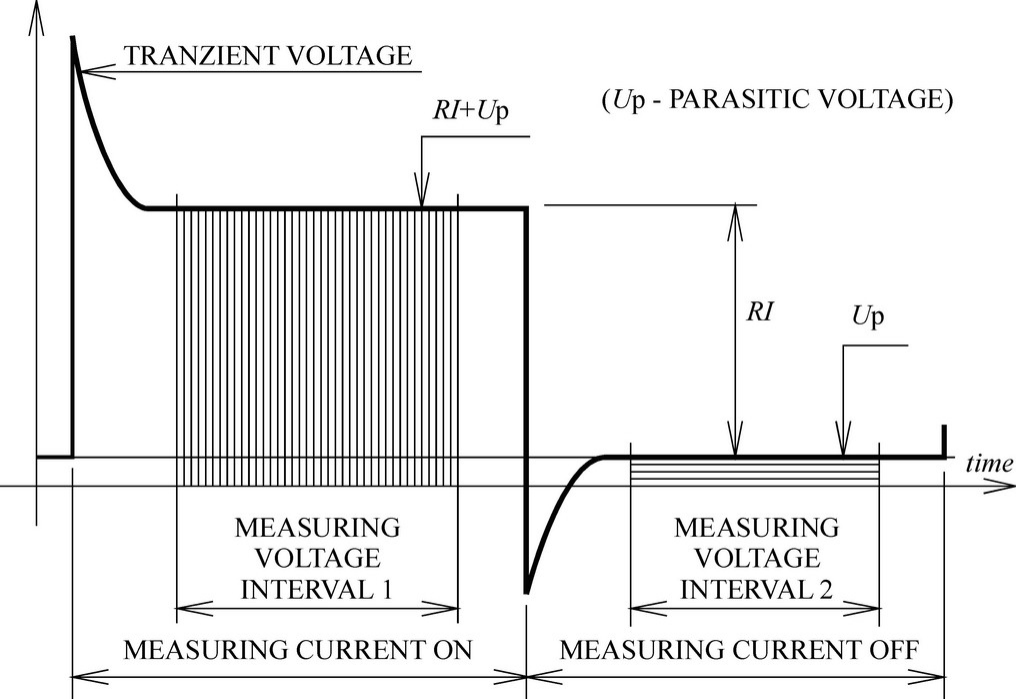
Parasitic voltage elimination.

There are several known methods for removing DC parasitic voltages. The main idea of DC voltage elimination is presented in Figure 2.

According to the figure, the voltages can be expressed as:

(3) $$U_{1}=R\times I+U_{P}$$

where *I* is the measuring current and *U_P_* parasitic DC voltage. When the measuring current is zero, the voltage should be:

(4) $$U_{2}=U_{P}$$

The measured voltage *U* is independent of parasitic voltage *U_P_*:

(5) $$U=U_{1}-U_{2}=R\times I$$

It is possible to use integrated operational amplifiers with chopper stabilized input voltage offset, such as ICL7650 [5]. The chopper stabilization includes the inner gates only, just to their input pins. The input offset voltages are reduced to 1 μV with temperature coefficient (TC) of 0.01 μV/°C, but sometimes there is a need to decrease the offset value below 1 μV. The improvement of the present solutions is, in fact, the main goal of design and realization of our electrical circuit with chopper elimination of DC parasitic voltage.

The principle circuit diagram of the low resistance comparator is shown in Figure 1. Both resistances (measured and reference), *R_X_* and *R_R_* are serially connected. The current circuit supplies them with a current of about 1 A. There is no need for high and long-term current stability. Since the current supply circuit is galvanically separated, the reference potentials are connected with an appropriate analog switch.

**Figure 3.**
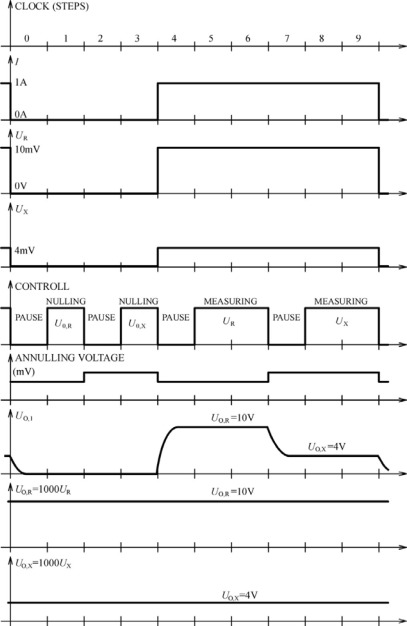
The controllers timing diagram.

The maximal value of resistances *R_X_* and *R_R_* for the chosen measuring range is about 10 *m*Ω and the *RI* voltages on their potential terminals are about 10 mV. Both voltages (*U_X_* and *U_R_*) are amplified by the same amplifier (G=1,000) and give maximal outputs of about 10 V (Figure 3). Besides the amplifier, there is a control circuit, circuit for chopper elimination of parasitic voltages (correction circuit) and output sample and hold circuits.

The controller switches the measuring current on and off and controls the functions of the voltage circuit analog switch. It is adjusted so that the duration of current pulse of 1 A is 60% of one controller cycle. During the remaining 40% of a cycle the current is switched off. While the measuring current is switched off, the amplifier's output should be zero, but parasitic voltage at the amplifier input occurs and it is amplified 1,000 times, as well.

Using correction and feed back circuits, this amplified voltage can be reduced to an acceptable value, below 10 μV. This is done for both resistances (*R_X_* and *R_R_*), sequentially. These correction voltages (annulling voltage, Figure 3.) are memorized on the corresponding capacitors and used while the measuring current is switched on. In a new cycle, when the measuring current is switched on again, both voltages (*U_X_* and *U_R_*) are amplified consecutively and the earlier memorized correction voltages are activated and included. The voltage without the component of parasitic unwanted voltage occurs for every resistor on the amplifier output. These two voltage outputs are separated by a demultiplexer and led to two independent sample and hold circuits, giving output voltages *U_OR_* and *U_OX_*, respectively. Their measurement or their difference gives the possibility of calculation of the resistance with very high accuracy using the above expressions.

The described solution for low resistance measurement allows a very useful possibility: to perform the measurement with a known value of resistor *R_R_*, then repeat the measurement with the same resistor (*R_X_* = *R_R_*). Let us call this a self-comparison mode (SCM). In this case, the measured value of both output voltages should be the same. But, in practice, these are slightly different. This difference is exactly equal to the comparator error. For the realized instrument, the average value of this difference for voltages of 10 V is less then 20 μV, or 2 ppm. The remaining AC voltages cause a dispersion of the measured values of up to 50 μV (5 ppm). For output voltages of about 10 V these voltage differences are acceptable.

In order to reach high measurement accuracy the instrument must have extremely high sensitivity. In such cases unwanted influences can occur. Some critical points of design, construction and practical realization are listed below:

- For a complete elimination of error caused by common mode rejection ratio (CMRR) input voltage, a special way of switching was applied. A third switch was added to connect the reference potential of the voltage circuit with the negative resistor potential terminal.
- The influence of transient processes was avoided by the use of appropriate length of dead time in controller cycle (pause, Figure 3).
- The leakage current of output sample and hold circuits should be extremely small because the voltage drop down mustn’t exceed 10 μV. The controller cycle is synchronized in such a way that the cycle step duration is a multiple of the main frequency period to be able to reach these conditions and thus eliminate the capacitive and inductive disturbances.
- In order to reduce the mains supply influence the controller cycle is synchronized with the mains supply frequency.
- Excellent quality operational amplifiers with very high open loop gain are used in the design.
- To achieve very high linearity, the complete amplification is realized with three-stage amplifiers with low gain (10 times each). The described solution allows the possibility of not only the resistance comparison., but with voltage ratio measurement (*U*_X_/*U*_R_) and high quality reference resistor R_R_ (standard resistor for example) it is possible to measure the resistance R_X_ with very high accuracy (milliohm meter).

## 3. Experimental Section

A 6½ digits A/D converter is embedded in the realized prototype instrument. The applied resistance of *R_R_* = 10 *m*Ω gives a measurement range of about 10 *m*Ω □ □ and 10 *n*Ω resolution. Excellent experimental results, presented in Table 1, were achieved in the SCM mode [6], where:

*- N* - number of independent repeated measuring attempts
- U_OX_, U_OR_ ≈ 10 V (corresponding R_X_, R_R_ =10 *m*Ω)
*- Mean* - error mean value
*- sd* - standard deviation
*- u* - standard deviation of the mean (Type A standard uncertainty)

**Figure 4.**
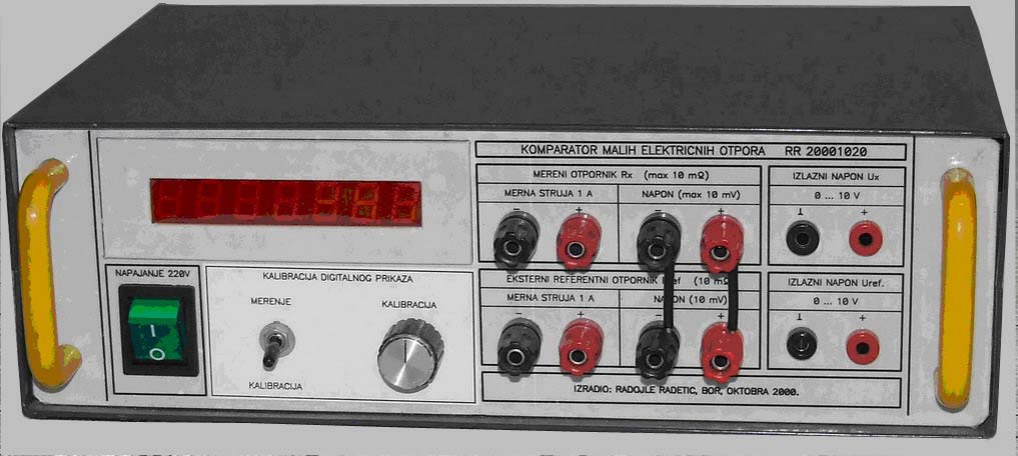
Low resistance comparator prototype.

The realized prototype instrument is shown in Figure 4. It has shown excellent results: for the measurement with a current of 1 A for a resistance of about 10 *m*Ω, the measurement deviation was no greater than 50 *n*Ω in every single attempt [6].

## 4. Conclusions

An improvement of the chopper method for elimination of parasitic voltages in a procedure of a small resistance comparison and measurement are presented in this paper. The realized instrument prototype had to overcome many practical difficulties. Some of them were solved empirically (i.e. choosing appropriate operational amplifiers) [7]. The nstrument is working as a prototype at this moment, but we have the intention of integrating it into laboratory equipment. All experiments were performed at the Department of Electrical Measuring of the Faculty of Technical Sciences in Novi Sad and confirmed by the official institution in Belgrade. Very high accuracy in resistance comparison and measurement is achieved (0.08 ppm for 1,000 independent measurement attempts and 0.26 ppm for 100 attempts). Although these are excellent results, there is room for further improvements. The main activities should be focused on design and realization of more different measurement ranges with the possibility of auto calibration. An interface for direct PC connection should be also developed, for the proposed comparator to become an intelligent instrument [8].

## Figures and Tables

**Table 1.** The experimental results.

***N***	***Mean (μV) (ΔU_O_=U_OX_-U_OR_)***	***ΔR (nΩ)***	***sd (μV)***	***u (ppm)***
100	11,2	11,2	25,6	0,26
1000	4,5	4,5	24,3	0,08
